# Editorial: Gastrointestinal infections in pediatric populations

**DOI:** 10.3389/fgstr.2026.1893817

**Published:** 2026-06-25

**Authors:** Sahil Khanna, Kanika Puri

**Affiliations:** 1 Division of Gastroenterology and Hepatology, Mayo Clinic, Rochester, MN, United States; 2 Division of Pediatric Gastroenterology, Hepatology and Nutrition, Indiana University School of Medicine, Indianapolis, IN, United States

**Keywords:** children, colitis, diarrhea, enteritis, fever, infections

This Research Topic was conceived to examine gastrointestinal infections in pediatric populations across the full continuum of risk, from environmental exposure and nutrition to the bedside management of complex hospitalized children. The resulting Research Topic makes clear that pediatric gastrointestinal infection is not a single clinical entity, but a network of interlocking biologic, social, and systems-level problems. In children, infectious diarrhea and related gastrointestinal morbidity remain tightly linked to malnutrition, unsafe water and sanitation, delayed access to care, and antimicrobial resistance (1, 2). The papers gathered here therefore span community prevention, caregiver knowledge, household transmission, hospital-based therapeutics, perioperative care, and antimicrobial-resistance surveillance.

A broad framing contribution comes from Verma et al., whose mini-review examines malaria and malnutrition in tribal areas of India. Although centered on malaria rather than a classic enteric pathogen, the paper is highly relevant to this Topic because it highlights the wider ecology in which pediatric gastrointestinal infections occur. The authors describe a vicious cycle in which undernutrition, anemia, poor WASH conditions, limited healthcare access, and repeated infection reinforce one another, while altered gut function and malabsorption further deepen nutritional vulnerability. Their call for integrated, district-level strategies that combine infectious disease control, nutrition-sensitive programs, surveillance, and improved access to care resonates strongly with the broader aims of this Research Topic.

At the community level, Shukla et al. show why prevention and early home management remain central. In a survey of mothers of children under five in Pune, awareness of diarrhea causes and oral rehydration solution (ORS) was generally good, and most respondents knew how to prepare ORS correctly. Yet important gaps persisted: fewer than one-third recognized the role of zinc supplementation, knowledge translated only weakly into practice; and only about one-quarter reported all three WHO-recommended home measures of ORS, increased fluids, and continued feeding. These findings remind us that public-health gains depend not simply on awareness, but on complete and consistent implementation of evidence-based care.

Two contributions address children whose gastrointestinal disease places them at particular hospital-based risk. Huo et al. used an ex vivo model of an E. coli-infected central venous catheter to compare treatment approaches relevant to children dependent on long-term venous access, including those with intestinal failure. Ceftriaxone produced the fastest antibacterial response, whereas taurolidine and ethanol-based approaches were also active but slower. Importantly, the study also highlights the potential of isothermal microcalorimetry as a rapid tool for assessing treatment response and refining catheter-salvage strategies. In a different but complementary setting, Li et al. examined 20% human serum albumin use after enteric anastomosis in children. Their propensity-matched retrospective analysis suggests that prolonged early postoperative albumin administration was associated with longer postoperative stay and more complications, supporting a more judicious approach to supportive therapies in fragile pediatric surgical patients.

A complementary brief report by Tang et al. brings the problem of recurrent enteric infection into the household. In a family cluster of pediatric nontyphoidal Salmonella, conventional culture detected the pathogen only at the index child’s first episode, whereas solid-phase enrichment identified additional culture-negative pediatric specimens and metagenomic sequencing detected Salmonella reads among household contacts. Genomic comparison supported a clonal household source. The report is important not only because it documents hidden transmission, but also because it shows how conventional culture, enrichment methods, and sequencing can function as complementary tools. For pediatric practice, it suggests that a more layered diagnostic strategy may be valuable when symptoms recur, but routine testing is unrevealing.

At the hospital level, Dinh et al. confront one of the most urgent threats in pediatric infectious disease: antimicrobial resistance. In a provincial pediatric hospital in Vietnam, carbapenem-resistant Enterobacterales colonization was found in 28.2% of hospitalized children, with prevalence rising after 48 hours of admission and with longer hospital stay, and with the highest burden in intensive care settings. The predominance of multidrug-resistant Escherichia coli, Klebsiella pneumoniae, and Enterobacter cloacae underscores the narrow therapeutic margins now faced by clinicians. This work shifts attention from treatment alone to colonization pressure, surveillance, targeted screening, and infection-prevention and control measures that are essential if subsequent invasive disease is to be prevented.

Taken together, the contributions in this Research Topic show that pediatric gastrointestinal infection cannot be understood through a single lens. It is shaped by poverty and nutrition, by what caregivers know and do at home, by transmission within households, by the technologies and therapeutics used in hospital care, and by the growing pressure of antimicrobial resistance. As editors, we see a practical agenda emerging from this Research Topic: integrate WASH, nutrition, and caregiver education with better microbiologic surveillance; use layered diagnostics to clarify hidden transmission and recurrent infection; link laboratory innovation to clinically actionable management strategies; and strengthen stewardship and infection prevention in the inpatient setting. Such an approach is more likely than isolated interventions to reduce the burden of gastrointestinal infections and related complications in children across diverse care settings.
